# Optimizing non-invasive brain stimulation for obsessive-compulsive disorder: a systematic review of protocol heterogeneity, personalized targets, and adjunctive efficacy

**DOI:** 10.1186/s12888-025-07723-1

**Published:** 2026-01-05

**Authors:** Mohammad Khosravi, Reza Khosravi, Amir Reza Tavassoli, Amir Jahanian-Najafabadi

**Affiliations:** 1 Department of Psychology, Shahid Beheshti University, Tehran, Iran; 2 Department of Psychology, Tehran University, Tehran, Iran; 3 Department of Cognitive Neuroscience, Bielefeld University, 33501 Bielefeld, Germany

**Keywords:** Repetitive transcranial magnetic stimulation, Obsessive-compulsive disorder, Supplementary motor area, Dorsolateral prefrontal cortex, Chronic disorder

## Abstract

Obsessive-Compulsive Disorder (OCD) is a chronic psychiatric disorder characterized by intrusive thoughts and repetitive behaviors, leading to significant distress and functional impairment. First-line treatments include selective serotonin reuptake inhibitors and cognitive-behavioral therapy, particularly exposure and response prevention (ERP). However, a substantial proportion of patients exhibit limited or no response to these interventions. Repetitive transcranial magnetic stimulation (rTMS) has shown potential in modulating dysfunctional cortical circuits implicated in OCD pathophysiology. Notably, the field achieved a major clinical milestone with the U.S. Food and Drug Administration (FDA) clearance in 2018 for Deep TMS (dTMS) targeting the medial prefrontal cortex (mPFC) and anterior cingulate cortex using the H7 coil, based primarily on positive randomized controlled trials. Despite promising findings, clinical studies on rTMS report heterogeneous outcomes, possibly due to variations in stimulation parameters, target regions, and study designs. These inconsistencies underscore the need for a comprehensive synthesis of current evidence. A comprehensive literature search was conducted across PubMed, ScienceDirect, and Scopus up to August 15, 2025. Eligible studies included adult OCD patients receiving multi-session rTMS protocols. Non-RCT, case reports, non-English studies, and non-primary literature were excluded. Risk of Bias Tool (RoB v1) was assessed using the Cochrane Collaboration tool. Forty-seven studies involving 1,632 participants were included. The most frequently targeted regions were the supplementary motor area (SMA) and dorsolateral prefrontal cortex (dlPFC). Low-frequency (1 Hz) stimulation was the most commonly applied protocol. While several studies demonstrated significant improvement in some of OCD symptoms, other studies reported minimal or no benefit. Methodological heterogeneity, particularly in stimulation parameters and target selection, precludes definitive conclusions. Notably, studies combining rTMS with pharmacological or psychotherapeutic interventions and those incorporating neurobiological predictors such as neuroimaging or genetic polymorphisms suggest potential for enhanced outcomes through personalized treatment strategies. Current evidence supports rTMS as a potentially effective adjunctive treatment for OCD, particularly when targeting the SMA and dlPFC. However, variability in methodological quality and treatment parameters underscores the need for standardized protocols and high-quality RCTs. Integrating neurobiological predictors and combining rTMS with established therapies may optimize treatment efficacy in future investigations.

## Introduction

Obsessive-Compulsive Disorder (OCD) is a chronic and debilitating psychiatric condition characterized by intrusive thoughts (obsessions) and repetitive behaviors (compulsions) that individuals feel compelled to perform [1, 2]. These symptoms often lead to significant distress and impairment in daily functioning [3]. Epidemiological studies indicate that OCD affects approximately 1% to 3% of the global population, with a lifetime prevalence of 2.3% among adults in U.S populations [4, 5]. The OCD typically manifests in late adolescence or early adulthood, although it can occur at any age [6]. The pathophysiology of OCD is complex and not fully understood, but neuroimaging and neuropsychological studies have implicated dysfunction in cortico-striato-thalamo-cortical (CSTC) circuits, particularly involving the orbitofrontal cortex, anterior cingulate cortex, and basal ganglia [7–9]. These neural circuits are believed to play a role in the regulation of intrusive thoughts and repetitive behaviors characteristic of OCD.

Standard treatment approaches for OCD include pharmacotherapy and psychotherapy. Selective serotonin reuptake inhibitors (SSRIs) are the first-line pharmacological treatment, often requiring higher doses and longer durations than those used for depression [10, 11]. Cognitive-behavioral therapy (CBT), particularly exposure and response prevention (ERP), is the most effective psychotherapeutic intervention [12, 13]. Despite these treatments, approximately 30–40% of patients do not achieve adequate symptom relief, highlighting the need for alternative or adjunctive therapies [14]. In recent years, non-invasive brain stimulation (NIBS) techniques, especially repetitive transcranial magnetic stimulation (rTMS) has emerged as a potential treatment for refractory OCD [15]. This field experienced a paradigm shift in 2018 when the U.S. Food and Drug Administration (FDA) granted marketing clearance for Deep Transcranial Magnetic Stimulation (dTMS) using the specialized H7 coil, targeting the medial prefrontal cortex (mPFC) and anterior cingulate cortex (ACC). This regulatory milestone, primarily driven by the pivotal clinical trial conducted by Carmi et al. [16], validated rTMS as an established therapeutic option, shifting the primary research focus from questioning its overall efficacy to optimizing specific stimulation protocols, coil types, and target circuits. rTMS uses magnetic fields to induce electric currents in specific cortical areas, aiming to normalize the dysfunctional neural circuits implicated in OCD [17]. Clinical studies investigating the efficacy of rTMS in OCD have yielded mixed results [18]. Some randomized controlled trials (RCTs) and meta-analyses report significant symptom improvement, particularly when targeting areas such as the dorsolateral prefrontal cortex (dlPFC) and supplementary motor area (SMA) [19]. However, other studies have found minimal or no benefit, and the heterogeneity in study designs, stimulation parameters, and patient populations complicates the interpretation of these findings [18].

While several systematic reviews and meta-analyses have been published, the field of rTMS for OCD is rapidly evolving. Our review represents the most comprehensive synthesis to date, including 47 randomized controlled trials, more than double the number of RCTs included in the most recent major meta-analyses (e.g. [14, 19]). Crucially, our search was current up to August 15, 2025, thereby incorporating the most recent 3–4 years of high-quality evidence (e.g. [20–22]) which significantly increases the statistical power and clinical relevance of our conclusions.

Building on previous syntheses, the primary novelty of this systematic review is threefold. First, it incorporates the significant volume of high-quality evidence published since 2022, dramatically increasing the pool of available RCTs. Second, it provides the first detailed comparative synthesis of emerging rTMS protocols and targets, including deep TMS (dTMS) targeting the mPFC/ACC and the latest findings on Theta Burst Stimulation (TBS). Third, and most importantly, our review uniquely focuses on the critical shift towards personalized medicine by synthesizing findings that integrate rTMS with neurobiological predictors (e.g., fMRI connectivity and genetic polymorphisms) and psychotherapeutic augmentation, offering actionable insights for defining optimal, individualized treatment strategies in refractory OCD.

Given the variability in study outcomes and the lack of consensus on optimal stimulation protocols, there is a clear need for a comprehensive systematic review. Such a review would synthesize current evidence on the efficacy of rTMS in treating OCD, and identify factors influencing treatment outcomes, and highlight areas requiring further research.

The primary objective of this systematic review is to evaluate the efficacy of rTMS, in the treatment of OCD and highlighting the gaps in the current literature to inform future research directions. Secondary objectives include: 1) Assessing the methodological quality of existing studies on rTMS for OCD. 2) Identifying optimal stimulation parameters (e.g., frequency, intensity, duration) and target brain regions associated with therapeutic benefits reported by prior research. 3) Exploring patient characteristics (e.g., age, symptom severity, treatment resistance) that may predict response to rTMS. By systematically reviewing and analyzing the available evidence, this study aims to provide clinicians and researchers with a clearer understanding of the potential role of rTMS in managing OCD, thereby contributing to the development of more effective, personalized treatment strategies.

## Method

### Inclusion and exclusion criteria

Studies were considered eligible for inclusion if they met all of the following criteria. First, the population consisted of adult participants aged 18 years or older with a formal clinical diagnosis of OCD. Second, the intervention involved the application of rTMS as a therapeutic modality. Third, the study design required a multi-session stimulation protocol, in which the same stimulation montage was applied across multiple treatment sessions, rather than a single-session experimental design. Fourth, only randomized controlled trials (RCTs) were eligible to ensure a high level of methodological rigor and to minimize bias.

Studies were excluded if they did not meet these standards, including case reports, single-case studies, and publications not written in English. Animal studies were not considered, as the review focused exclusively on human clinical populations. Additionally, systematic reviews, meta-analyses, conference abstracts, and other non-primary research articles were excluded to maintain focus on original experimental and clinical data. Finally, studies that were non-randomized or uncontrolled were excluded to avoid potential confounding effects and to ensure that findings reflected the causal impact of rTMS interventions.

### Search strategy

**Fig. 1:**
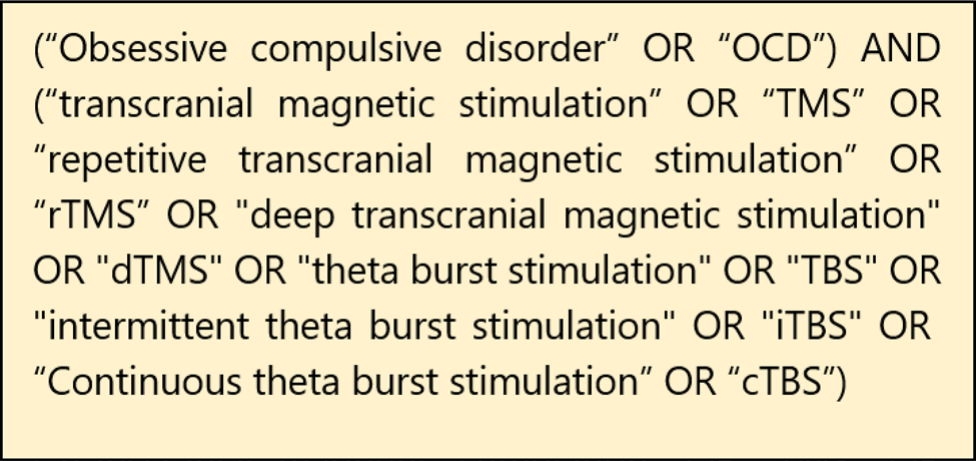
Syntaxes used for systematic search

A comprehensive literature search was conducted in four major electronic databases: PubMed, ScienceDirect, Web of Science, and Scopus, covering the period from conception to 15 August 2025. The search strategy combined Medical Subject Headings (MeSH) terms and keywords related to OCD and rTMS. The complete search syntaxes for each database are presented in Fig. 1. The search retrieved 2568 records. All citations were imported into EndNote (Clarivate Analytics) for reference management, where 1174 duplicates were automatically removed. The remaining 1374 records were uploaded into Rayyan, an online systematic review platform, for screening.

### Screening, study selection and data extraction

**Fig. 2:**
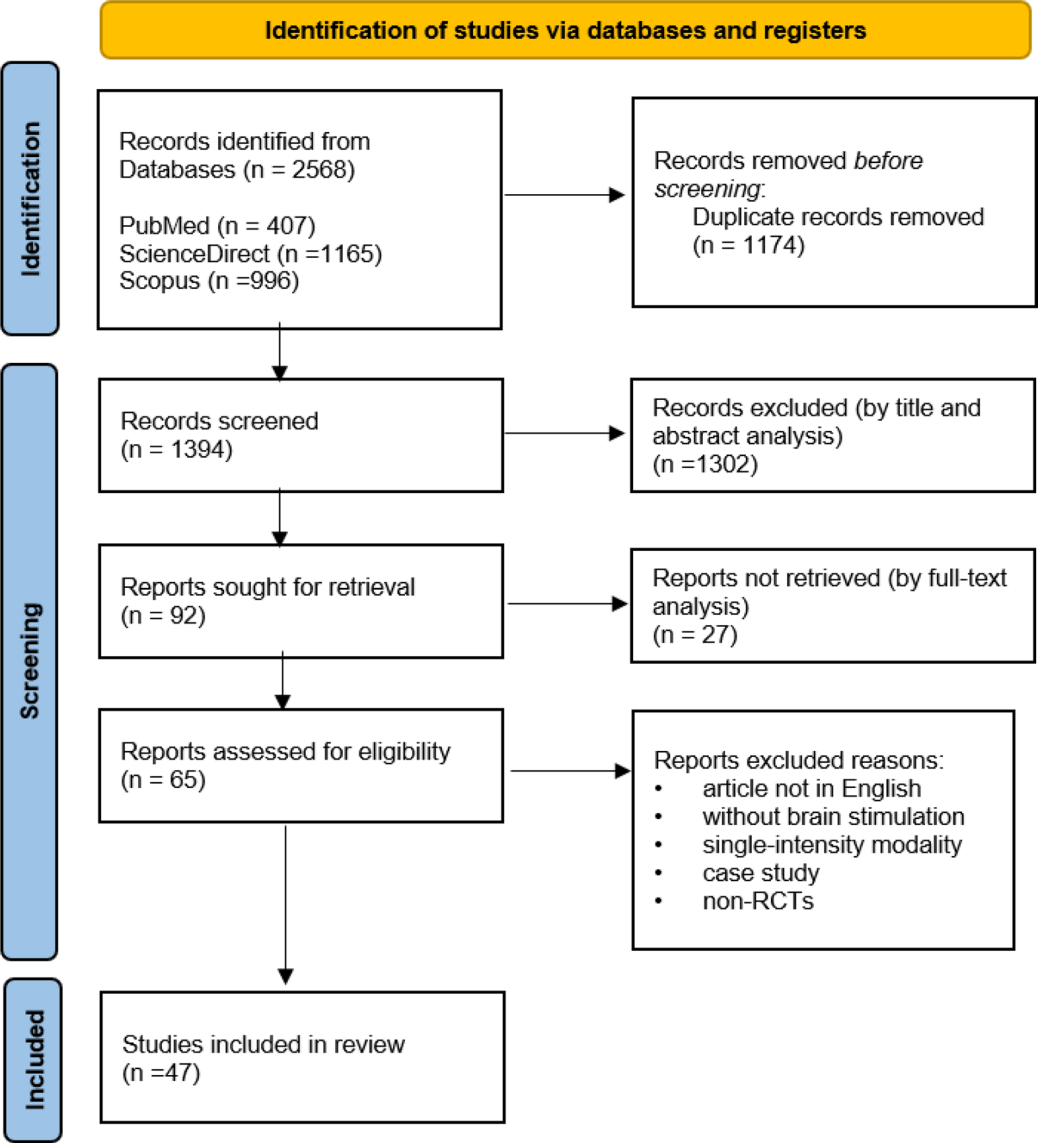
The PRISMA flowchart of the systematic review process

Screening and selection were conducted in multiple stages, as illustrated in the PRISMA flow diagram (Fig. 2). Initially, the titles and abstracts of the 1374 records were screened independently by two reviewers (RK, and ART) using the predefined inclusion and exclusion criteria. Articles deemed potentially relevant proceeded to full-text review, also conducted independently by the same reviewers. Discrepancies in study selection at either stage were resolved through discussion. When consensus could not be reached, the other reviewers (MK, AJN) acted as arbitrator to make the final decision. Following full-text assessment, 82 studies were evaluated in detail, and 47 met all eligibility criteria for inclusion in the final analysis. The detailed process of screening and selection is depicted in Fig. 2, highlighting the progressive narrowing of the dataset and the systematic application of inclusion and exclusion criteria. Data from included studies were extracted into a standardized Excel sheet. Recorded variables included study identification (title, authors, year), stimulation parameters (frequency, intensity, pulses per session, session duration, total sessions), study design, participant characteristics (sample size, age, sex), target population, outcome measures, and main findings related to the effects of rTMS on OCD symptoms.

### Risk of bias

The methodological quality of the included studies was assessed using the Cochrane Risk of Bias Tool (version 1), which evaluates seven domains: random sequence generation, allocation concealment, selective reporting, blinding of participants and personnel, blinding of outcome assessment, incomplete outcome data, and other potential sources of bias. Each domain was rated as low risk, high risk, or unclear risk in accordance with Cochrane RoB v1 guidelines.

Overall risk of bias judgments was determined based on Cochrane criteria, whereby the presence of any high-risk domain resulted in an overall judgment of high risk, and the presence of any unclear domain (without high risk) resulted in an overall judgment of unclear risk. Only studies rated as low risk across all domains were assigned an overall judgment of low risk.

Risk of bias assessments was conducted independently by two reviewers. Discrepancies were resolved through discussion, and when consensus could not be reached, the other reviewers acted to arbitrate.

Following the removal of open-label trials from the dataset, most included studies met the criteria for an overall low risk judgment. The final domain-specific and overall ratings are summarized in Fig. 2.

### Results

Altogether, this systematic review included a total of 47 studies, which assessed a sample of 1632 participants. The mean age of the participants ranged from 23.1 to 47.25 years old, and the sample sizes varied from 10 to 63. Total number of sessions ranged from 5 to 30 sessions Table 1.

**Table 1:**
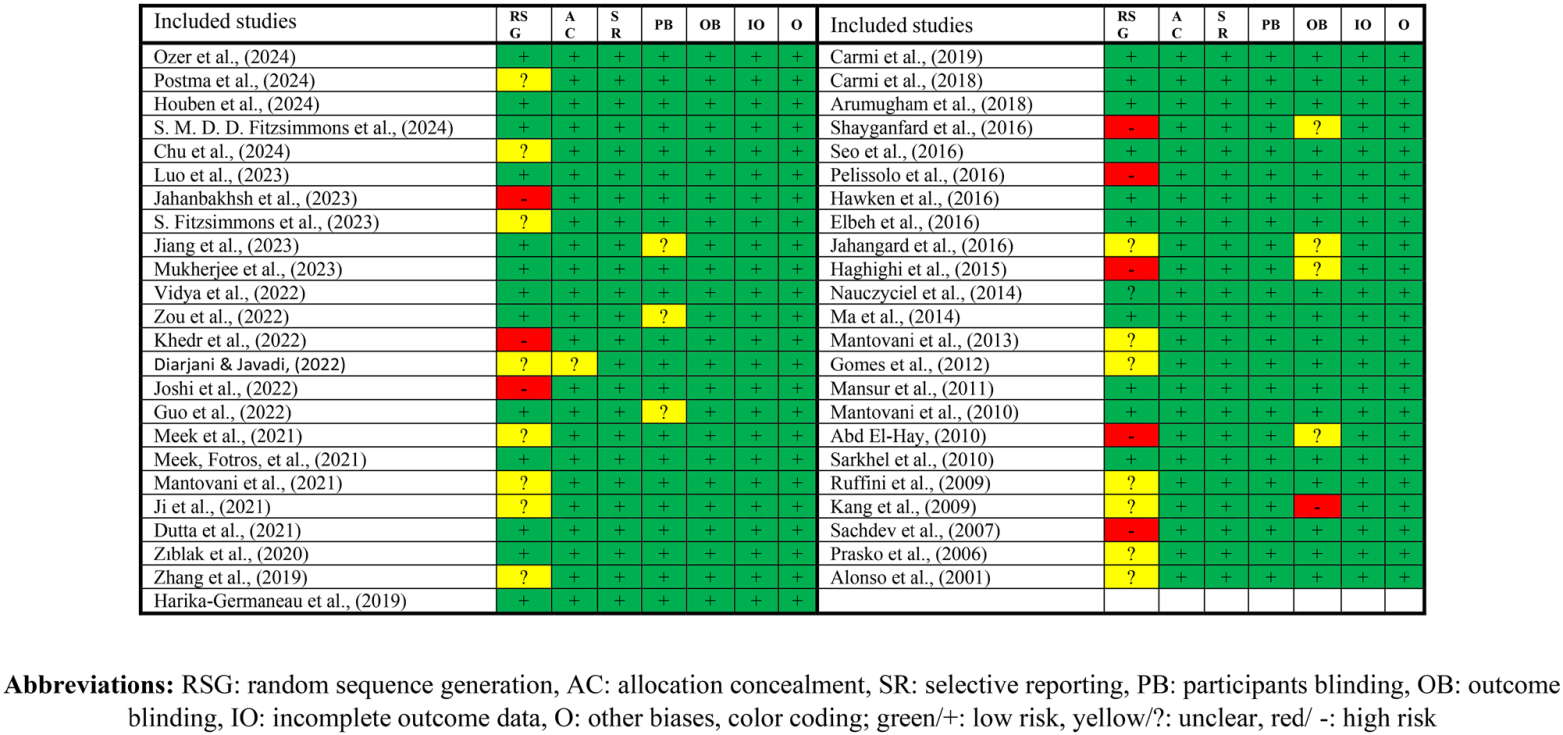
Risk of bias in the included studies

The most frequently targeted brain region was SMA (19 studies), followed by the dlPFC (14 studies). Five studies targeted both dlPFC and SMA simultaneously, one study both dlPFC and OFC and 3 studies targeted both mPFC and ACC. The orbitofrontal cortex (OFC) was targeted in 4 studies. The dorsal anterior cingulate cortex (dACC) was targeted in 1 study. The insula and amygdala were investigated as part of connectivity analyses but not directly stimulated as primary targets. The studies examined a range of stimulation intensities, with the most common being 1 Hz (25 studies) and 10 Hz (7 studies). Some studies also investigated 20 Hz (7 studies) and 8–12 Hz (1 study) and one study used both 1/10 Hz and another study used 6 + 1 Hz and 5 studies used TBS (50 Hz bursts at 5 Hz). The most common duration was 20 minutes, reported in 18 studies.

**Fig. 3:**
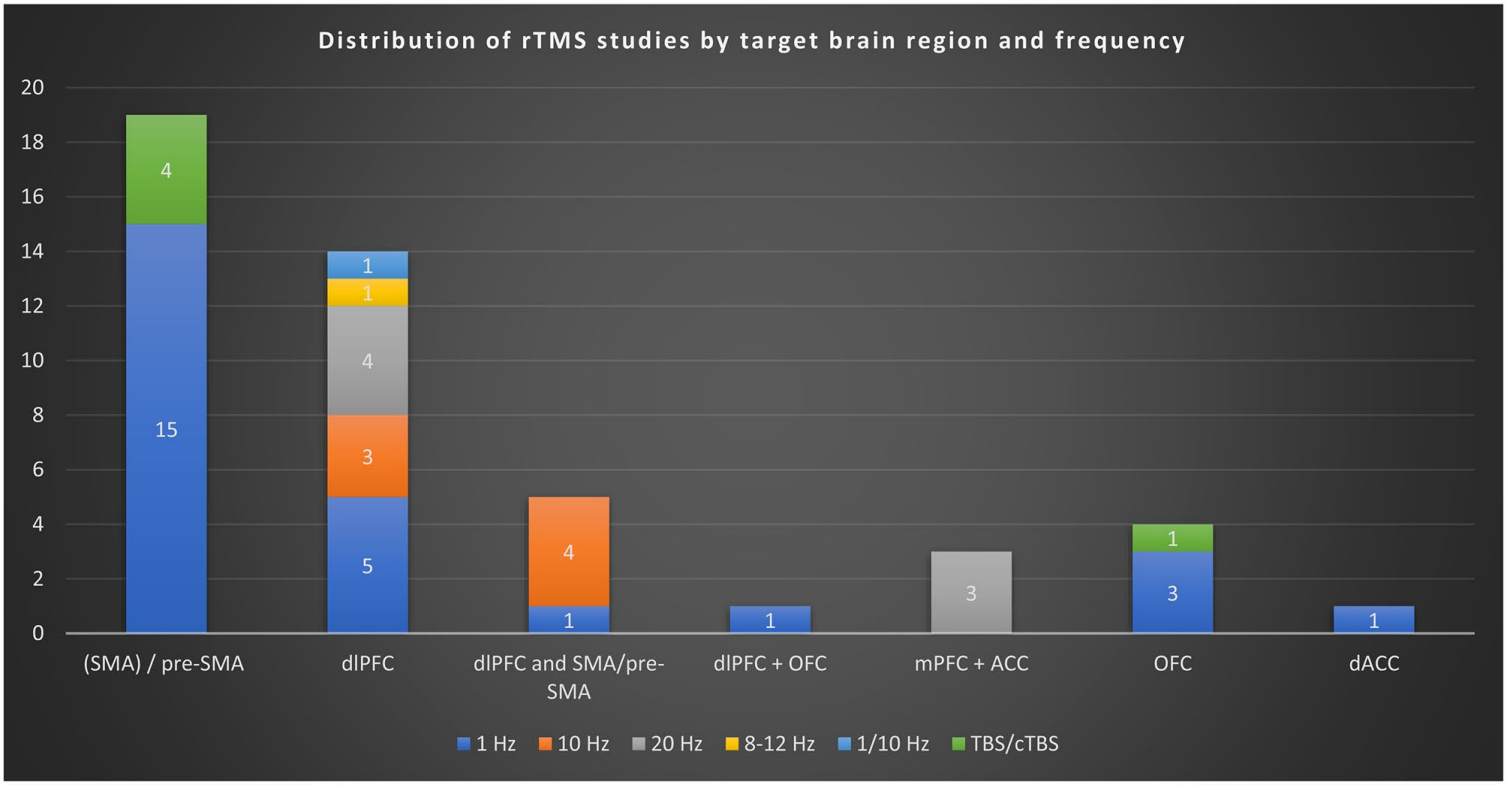
Distribution of rTMS studies in OCD by target brain region and stimulation frequency. The most frequently targeted region was the supplementary motor area (Sma)/pre-SMA (19 studies), followed by the dorsolateral prefrontal cortex (dlPFC) (14 studies). Combined stimulation of dlPfC and Sma/pre-SMA was investigated in five studies, while four studies targeted the orbitofrontal cortex (OFC), three targeted the medial prefrontal cortex (mPFC) with the anterior cingulate cortex (acc), one targeted both dlPfC and Ofc, and one targeted the dorsal anterior cingulate cortex (dACC). Frequencies included 1 Hz, 10–20 Hz, 8–12 Hz, 1/10 Hz, and theta burst stimulation (tbs). Bars represent the number of studies per region, stacked by stimulation frequency

The features of each study are summarized in Table 2, and the distribution of studies by target brain region and stimulation frequency is illustrated in Fig. 3. In the following sections, the results are organized according to the primary brain region targeted, including the SMA, dlPFC, OFC, and ACC and further grouped by the stimulation frequency applied (e.g., 1 Hz, 10 Hz, 20 Hz, and theta-burst stimulation protocols).

**Table 2:** The results of included rTMS studies

Study	Design	N (I, C)	Age Mean (SD), Gender (M:F)	F (Hz), Intensity (% of MT), No. of pulses, D(min), Sessions(n)	Region	**Measures**^**2**^	Main results
Ozer et al. [23]	Randomized, double-blind, sham-controlled	29 (14, 15)	34.5 (13.3), 5:9	20, 110, 2000, 17.97, 30	mPFC + ACC	Y-BOCS, DOCS, HAM-A, HAM-D, CGI	Active dTMS with double-cone coil twice daily for 3 weeks significantly reduced Y-BOCS and HAM-A scores compared to sham. No significant reduction in symmetry-related OCD symptoms. 35% response in active vs. 6.7% in sham. Treatment well tolerated with mild side effects.
Postma et al. [22]	Randomized, double-blind, sham-controlled	61 (18/23, 19)	37.25 (12.84), 21:39	10, 110, 3000, 20, 16	dlPFC, pre-SMA, vertex	YBOCS, BDI, BAI	pretreatment fMRI activation and connectivity during planning and inhibition processes predicted clinical response to specific rTMS conditions in OCD. Lower precuneus activation in the DLPFC group and lower insular-amygdalar connectivity in the preSMA group were associated with symptom reduction.
Houben et al. [21]	Randomized, double-blind, sham-controlled	61 (19/23, 19)	36.97 (12.93), 22:39	10, 110, 3000, 20, 16	dlPFC, pre-SMA, vertex	YBOCS, BDI, BAI	Higher pretreatment activation of the right amygdala in response to OCD-related stimuli was positively associated with better treatment outcomes across all rTMS groups. Whole-brain analyses also identified additional task-relevant regions linked to treatment response, while greater dorsal anterior cingulate cortex activation to fear-related stimuli was negatively associated with treatment outcomes.
S. M. D. D. Fitzsimmons et al. [24]	Randomized, double-blind, sham-controlled	61 (19/23, 19)	36.97 (12.93), 22:39	10, 110, 3000, 20, 16	dlPFC, pre-SMA, vertex	YBOCS, BDI, BAI	rTMS to the preSMA and DLPFC combined with ERP significantly reduced OCD symptoms, with symptom improvement linked to decreased activation in planning and error-processing networks. However, no differences in symptom reduction were found between treatment groups.
Chu et al. [20]	Randomized, double-blind, sham-controlled	36 (16, 20)	31.61 (ns), 17:19	1, ns, ns, ns, 10	pre-SMA	Y-BOCS, HAM-A, HAM-D	Specific genetic polymorphisms (RS4680, RS16965628, and RS1019385) were identified as potential biomarkers for predicting treatment response to rTMS and SSRIs in OCD. These findings suggest a role for genetic profiling in optimizing precision treatment for OCD.
Luo et al. [25]	Randomized, double-blind, sham-controlled	52 (29, 23)	26.34 (6.36), 32:20	1, 110, 2000, 35, 20	bilateral SMA	Y-BOCS, HAM-A, HAM-D, BDI, BAI, GAF	rTMS combined with medication significantly reduced OCD (*p* = 0.038), anxiety (*p* = 0.012), and depression (*p* < 0.013) symptoms while increasing pACC tNAA levels (*p* = 0.030). Anxiety reduction correlated with pACC glutamate changes (*r* = −0.434, *p* = 0.02), suggesting rTMS efficacy is linked to neurometabolic modulation.
Jahanbakhsh et al. [26]	Randomized, double-blind, sham-controlled	30 (15, 15)	34.53 (9.75), 10:20	1, ns, 1200, 20, 15	left dlPFC	Y-BOCS	Adjunctive low-frequency (1 Hz) rTMS over the left DLPFC significantly reduced Y-BOCS scores in treatment-refractory OCD patients (*p* < 0.05), with effects lasting up to six months. The combination of antipsychotic and serotonergic agents further enhanced treatment response (*p* < 0.05).
S. Fitzsimmons et al. [27]	Randomized, double-blind, sham-controlled	61 (19/23, 19)	36.97 (12.93), 22:39	10, 110, 3000, 20, 16	dlPFC, pre-SMA, vertex	YBOCS, BDI, BAI	rTMS over DLPFC and preSMA combined with exposure and response prevention significantly reduced OCD severity (*p* < 0.001) in all groups, with a 57.4% response rate. Symptom improvement was linked to decreased planning-related activation in the DLPFC group and decreased error-related activation in the preSMA group, though no overall differences were found between treatment protocols.
Jiang et al. [28]	Randomized, single-blind, sham-controlled	45 (23, 22)	26.61 (6.99), 26:19	TBS (50 Hz bursts at 5 Hz), 80, 18,000/day, ns, 5	right pre-SMA	Y-BOCS, OCI-R, HAMD, HAMA, TMT, SCWT, DS	ahTBS significantly reduced Y-BOCS scores after 5 days, similar to 1-Hz rTMS. Trend for higher response rate in ahTBS group (61% vs. 32%), but not statistically significant. No adverse cognitive effects or serious side effects reported.
Mukherjee et al. [29]	Randomized, double-blind, sham-controlled	26 (13, 13)	ns, 13:13	TBS (50 Hz bursts at 5 Hz), 80, 1800/day, ns, 30	SMA	Y-BOCS, CGI-S, CGI-C, HAM-D, HAM-A	Active cTBS group showed significant group × time interaction for Y-BOCS obsession, compulsion, total, CGI-S, CGI-C, and HAM-D with large effect sizes. Improvements were significant at both week 3 and week 8. No significant difference in CGI-C response rates or Y-BOCS responders. Treatment was well tolerated.
Vidya et al. [30]	Randomized, double-blind, sham-controlled	30 (15, 15)	30.86 (12.93), 12:18	6 + 1, 80 + 100, 1800, 30, 10	SMA	Y-BOCS, HAM-D, HAM-A, CGI-I	Both groups improved over time, but active priming rTMS (6+1 Hz) produced significantly greater reduction in Y-BOCS compulsion, HAM-D, HAM-A, and CGI-I scores. No significant effect on Y-BOCS obsession or total scores. No serious adverse events observed.
Zou et al. [31]	Randomized, controlled	63 (32, 31)	23.1 (9.6), 38:25	1, 100, 1160, 26, 5	bilateral SMA	Y-BOCS, HAM-A, HAM-D, SCL-90, AAQ-II, CFQ	Sertraline combined with either ACT or rTMS significantly reduced OCD (*p* < 0.05), anxiety, and depression symptoms (*p* < 0.001), with no difference in overall efficacy between groups. However, ACT uniquely improved psychological flexibility (AAQ-II, CFQ) from 4 weeks post-treatment to follow-up (*p* < 0.01), whereas rTMS showed no such effect.
Khedr et al. [32]	Randomized, double-blind, sham-controlled	60 (20/20,20)	35.4 (10.1), 28:38	1, 120, 1500, ns, 10	R-dlPFC/R-OFC	Y-BOCS, HAM-A, BDI, CGI-S, MMSE, MoCA	Both right DLPFC and OFC rTMS significantly improved OCD symptoms, outperforming the sham group, with no difference between the active treatments. Lower baseline Y-BOCS and fewer comorbidities were predictors of better treatment response.
Diarjani & Javadi [33]	double-blind, sham-controlled	30 (15, 15)	39.3 (ns), 8:22	1, 110, 1200, 20, 15	left dlPFC	Y-BOCS	Both the rTMS and sham groups showed reduced Y-BOCS scores, with no significant difference between them (*p* = 0.82). After 15 sessions, OCD symptoms improved in both groups, but rTMS did not show a significant therapeutic effect, and no serious side effects were reported.
Joshi et al. [34]	Randomized, double-blind, sham-controlled	24 (13, 11)	31.85 (7.56), 14:10	1, 100, 1600, ns, 20	SMA	Y-BOCS	rTMS significantly reduced OCD symptoms, including both obsessions and compulsions, compared to sham treatment. The rTMS group showed a substantial improvement (*p* = 0.001), with a large effect size (Cohen’s d = 1.6), and only mild side effects were reported. These findings support rTMS as a safe and effective early augmentation strategy for drug-naïve OCD patients.
Guo et al. [35]	Randomized, single-blind, sham-controlled	50 (26, 24)	32.75 (8.99), 33:17	TBS (50 Hz bursts at 5 Hz), 110, 1200, 0.8, 20	bilateral SMA	Y-BOCS, HAMD24, HAMA14, OBQ44	No significant group difference in Y-BOCS at week 4 or 8. Significant improvement in HAMD and HAMA scores in the active group at week 4, not maintained at follow-up. No significant effects on cognitive tasks. cTBS was safe and well tolerated.
Meek et al. [36]	Randomized, double-blind, sham-controlled	22 (11, 11)	39.2 (11.4), 10:12	1, 100, 1200, 20, 20	bilateral SMA	Y-BOCS, BDI, BAI	Both groups showed improvement in Y-BOCS, but only active group maintained significant improvement at 1-month follow-up. Significant reduction in BDI scores in active group at endpoint. rTMS over SMA may be effective for treatment-resistant OCD.
Meek, Fotros, et al. [36]	Randomized, double-blind, sham-controlled	20 (10, 10)	41.65 (14.6), 10:10	1, 120, 1200, 20, 20	dACC	Y-BOCS, BDI, BAI, MoCA	Active group showed significant Y-BOCS improvement sustained at 3-month follow-up. Improvements in Flanker task RTs (congruent, incongruent, post-correct, error-reporting, and correction) only in active group. No adverse events reported. rTMS over dACC improved error-monitoring and clinical symptoms.
Mantovani et al. [37]	Randomized, double-blind, sham-controlled	18 (9, 9)	39.6 (9.4), 11:7	1, 110, 3600, 60, 20	bilateral SMA	Y-BOCS, CGI	MRI-guided double-daily rTMS to the SMA reduced OCD symptoms by 25% (*p* = 0.005) with sustained benefits at three months. Treatment was safe and decreased rsFC between the SMA and subcortical regions, suggesting a neural basis for symptom improvement.
Ji et al. [38]	Randomized, double-blind, sham-controlled	37 (20, 17)	27.75 (1.58), 27:10	1, 110, 7500, 30, 14	right pre-SMA	Y-BOCS, HARS, HADS	Personalized rTMS targeting the preSMA improved OCD symptoms (*p* = 0.019) and reduced target network connectivity, with predictive baseline connectivity patterns (*r* = .60, *p* = 0.009). These findings suggest that symptom relief is linked to decreased connectivity strength in OCD-related networks.
Dutta et al. [39]	Randomized, double-blind, sham-controlled	33 (18, 15)	29.5 (10.3), 16:17	TBS (50 Hz bursts at 5 Hz), 80, 1200, 0.67, 10	left OFC	Y-BOCS, HAM-D, HAM-A, CGI-S	Significant group × time interaction found for Y-BOCS, HAM-D, HAM-A, and CGI-S; but when controlling for HAM-A and HAM-D changes, only HAM-A and CGI-S remained significant. Response rate for OCD: 16.6% in active vs. 6.7% in sham. cTBS was well tolerated with only transient mild headache.
Zıblak et al. [40]	Randomized, double-blind, sham-controlled	34 (19, 15)	41.47(10.23), 11:23	1, ns, ns, ns , 20	right OFC	Y-BOCS, HAM-A, HAM-D	no significant differences between the rTMS and placebo groups in demographic factors or Y-BOCS score changes after 4 weeks of treatment. The decrease in Y-BOCS scores was under 35% for all patients, suggesting that rTMS targeting the OFC is not an effective add-on treatment for OCD.
Zhang et al. [41]	Randomized, double-blind, sham-controlled	49 (25, 24)	36 (15.2), 29:20	1, 100, 1200, 20, 20	pre-SMA	Y-BOCS, HAM-A, HAM-D	rTMS significantly reduced OCD symptoms, with greater improvement in patients with the LL genotype of 5-HTTLPR (*p* < 0.05), while S allele carriers showed no significant response. These findings suggest 5-HTTLPR as a potential biomarker for rTMS efficacy in OCD.
Harika-Germaneau et al. [42]	Randomized, double-blind, sham-controlled	28 (14, 14)	47.25 (11.7), 13:15	TBS (50 Hz bursts at 5 Hz), 70, 600, 0.67, 30	pre-SMA	Y-BOCS, CGI-S, MADRS, BAS, GAF	No significant difference between active and sham cTBS groups in Y-BOCS reduction at week 6 or week 12. Responder rates were 28.4% (active) vs. 35.7% (sham). No significant effects on secondary outcomes. cTBS was well tolerated with only mild headache reported.
Carmi et al. [16]	Randomized, double-blind, sham-controlled	94 (47, 47)	38.8 (11.8), 40:54	20, 100, 2000, ns, 29	mPFC + ACC (dTMS, H7 coil)	Y-BOCS, HAM-D, SDS, CGI-I, CGI-S	Active dTMS significantly reduced Y-BOCS scores compared to sham at posttreatment and 1-month follow-up. Response rate (≥30% Y-BOCS reduction) was 38.1% (active) vs. 11.1% (sham). CGI-I and CGI-S also improved significantly. Treatment well tolerated with no serious adverse events. Personalized symptom provocation used before each session
Carmi et al. [43]	Randomized, double-blind, sham-controlled	30 (16, 14)	33.3 (7.7), 15:15	20, 100, 2000, ns, 25	mPFC + ACC (dTMS, H7 coil)	Y-BOCS, HAM-D, CGI-S, CGI-I	Active dTMS group showed significantly greater Y-BOCS reduction than sham. Response rate (≥30% Y-BOCS reduction) was 43.8% vs. 7.1% at post-treatment, and 45.5% vs. 7.7% at 1-week follow-up. CGI-I also significantly improved. Treatment associated with increased ERN theta activity correlated with symptom reduction. No serious adverse events. LF group was dropped after interim analysis.
Arumugham et al. [44]	Randomized, double-blind, sham-controlled	36 (19, 17)	27.74 (7.88), 8:28	1, 100, 1200, 20, 18	pre-SMA	Y-BOCS, HAM-A, HAM-D	no significant difference in OCD symptoms or other measures between the active and sham rTMS groups after 3 weeks of treatment. These results suggest that low-frequency rTMS over the pre-SMA may not be effective as an augmentation for partial/poor responders to pharmacotherapy.
Shayganfard et al. [45]	Randomized, single-blind, sham-controlled	10 (5, 5)	33.5(9.55), 4:6	20, 100, 750, 25, 10	bilateral dlPFC	Y-BOCS, CGI	rTMS reduced OCD symptom severity but had no significant effect on executive functions compared to sham treatment. Executive function performance improved over time, independent of rTMS. These findings support rTMS as a symptom-relief intervention while highlighting the need for further research on its cognitive effects.
Seo et al. [46]	Randomized, double-blind, sham-controlled	27 (14, 13)	35.4 (11.2), 14:13	1, 100, 1200, 20, 15	right dlPFC	Y-BOCS, HAMD, HAMA, BDI, CGI-S, HDRS	rTMS significantly reduced OCD and depression symptoms compared to sham treatment after three weeks. Repeated-measures ANOVA showed a significant time × group interaction on Y-BOCS, HDRS, and CGI-S scores. No serious adverse effects were reported, supporting rTMS as a potential treatment for resistant OCD.
Pelissolo et al. [47]	Randomized, double-blind, sham-controlled	36 (20, 16)	41.5 (10.7), 17:23	1, 100, 1500, 26, 20	pre-SMA	Y-BOCS, CGI-S, BAS	1-Hz rTMS over the presupplementary area did not significantly improve OCD symptoms compared to sham treatment after four weeks. Responder rates were low and did not differ between groups (10.5% vs. 20%, *p* = 0.63). These findings suggest that low-frequency rTMS in this region is ineffective for severe, medication-resistant OCD.
Hawken et al. [48]	Randomized, double-blind, sham-controlled	22 (10, 12)	33.5 (12.4), 11:11	1, 110, ns, 20, 25	bilateral pre-SMA	Y-BOCS, CGI, HDRS	six weeks of bilateral low-frequency rTMS over the sensorimotor area significantly reduced OCD symptoms compared to sham treatment, with effects persisting for six weeks post-treatment. Y-BOCS scores showed a clinically meaningful decline in the active group. Further research is needed to confirm the generalizability and duration of these effects.
Elbeh et al. [49]	Randomized, double-blind, sham-controlled	45 (15/15, 15)	27.1 (4.5), 30:15	1/10, 100, 2000, ns, 10	right dlPFC	Y-BOCS, HAMA, CGI-S	1 Hz rTMS over the right DLPFC significantly improved OCD and anxiety symptoms compared to sham and 10 Hz rTMS. A significant time × group interaction was observed for Y-BOCS and HAM-A (*p* = 0.001 and 0.0001, respectively), with greater clinical benefits in the 1 Hz group. These results suggest that 1 Hz rTMS may be a promising treatment for OCD.
Jahangard et al. [50]	Randomized, single-blind, sham-controlled	10 (5, 5)	33.10 (7.06), 7:3	20, 100, 750, 50, 20	bilateral dlPFC	Y-BOCS, CGI	rTMS significantly reduced OCD symptoms and improved cognitive performance, including verbal processing speed and flexibility, in patients with refractory OCD. These improvements were observed under the treatment condition but not under the sham condition, supporting rTMS as an effective treatment.
Haghighi et al. [51]	Randomized, single-blind, sham-controlled	21 (10, 11)	35.86 (11.02), 12:9	20, 100, 1500, 50, 10	bilateral dlPFC	Y-BOCS, CGI	rTMS significantly reduced OCD symptom severity compared to sham treatment in patients with treatment-resistant OCD. Both self- and expert-rated assessments showed improvements, with full and partial responses observed only in the rTMS group. These findings support rTMS as a promising intervention for treatment-resistant OCD.
Nauczyciel et al. [52]	Randomized, double-blind, sham-controlled	19 (9,10)	39 (24.56), 4:15	1, 120, 1200, ns, 10	right OFC	Y-BOCS, CGI, MADRS	Low-frequency rTMS over the right OFC reduced OCD symptoms, with a greater but nonsignificant improvement compared to sham (*p* = 0.07). PET scans showed decreased OFC metabolism after stimulation, supporting its role as a key target for rTMS in OCD treatment.
Ma et al. [53]	Randomized, double-blind, sham-controlled	46 (25,21)	27.17(8.97), 13:8	8–12, 648–872,20,10	bilateral dlPFC	YBOCS, HRSD, HAMA, CGI	Alpha EEG-guided rTMS over the DLPFC significantly reduced OCD and anxiety symptoms compared to sham treatment, with improvements persisting at follow-up. Depression symptoms showed a delayed response, suggesting secondary effects from OCD and anxiety relief.
Mantovani et al. [54]	Randomized, double-blind, sham-controlled	18 (9,9)	39.7 (8.6), 11:7	1, 100, 1200, 20, 20	bilateral pre-SMA	Y-BOCS, Y-BOCS-SR, CGI-S	Low-frequency rTMS over the SMA in OCD patients increased right hemisphere RMT and SICI, correlating with symptom improvement. Normalization of baseline RMT asymmetry was also linked to better clinical outcomes, supporting SMA rTMS as a modulator of dysfunctional motor circuits in OCD.
Gomes et al. [55]	Randomized, double-blind, sham-controlled	22 (12, 10)	36.4 (12.7), 9:13	1, 100, 1200, 20, 10	pre-SMA	Y-BOCS, Y-BOCS –SR, Ham-D–24, Ham-A–14, BDI–II, BAI, CGI–S	bilateral SMA rTMS significantly reduced OCD symptoms, with a 35% Y-BOCS reduction at 14 weeks compared to 6.2% in the sham group. Response rates were 41% for active and 10% for sham treatment.
Mansur et al. [56]	Randomized, double-blind, sham-controlled	27 (ns)	40.6 (13), 14:13	10, 110, 2000, ns, 30	right dlPFC	YBOCS, CGI, HAM-A, HAM-D, Short-form Health Survey	no significant difference between active and sham rTMS over the right dlPFC in treatment-resistant OCD patients (*p* = 1.00). While there was a significant effect of time on YBOCS scores (F = 7.33, *p* = 0.002), no significant group or group × time interaction was observed, indicating that active rTMS did not outperform sham in reducing symptoms or clinical severity.
Mantovani et al. [57]	Randomized, double-blind, sham-controlled	18 (9, 9)	39.6 (9.4), 11:7	1, 100, 1200, 20, 20	pre-SMA	YBOCS, YBOCS-SR, HAM-D, HAM-A, BDI, Zung-SAS, CGI-S,	medication-resistant OCD patients receiving 1-Hz rTMS to the supplementary motor area (SMA) showed a 67% response rate with active rTMS, compared to 22% with sham. Active rTMS resulted in a 25% reduction in YBOCS scores and normalized hemispheric laterality, supporting further research on rTMS for treatment-resistant OCD.
Abd El-Hay [58]	Randomized, single-blind, sham-controlled	40 (20, 20)	28.9 (5.7), 17:23	20, ns, ns, ns, 15	left dlPFC	Y-BOCS,	rTMS was not effective as monotherapy for OCD, but it significantly improved symptoms when used as an add-on treatment for patients with poor SSRI responses, reducing hyperexcitable brain circuits.
Sarkhel et al. [59]	Randomized, double-blind, sham-controlled	42 (21, 21)	30.7 (7.2), 23:19	10, 110, ns, ns, 10	right dlPFC	YBOCS, HAM-A, HAM-D, CGI-S	For YBOCS scores, no treatment-over-time effect was found (*p* = 0.262). Significant treatment-over-time effects were observed for HAM-D (*p* = 0.035, η^2^ = .158) and HAM-A (*p* = 0.01, η^2^ = .211). High-frequency right prefrontal rTMS showed no significant effect on OCD but modestly improved depressive symptoms.
Ruffini et al. [60]	Randomized, double-blind, sham-controlled	23 (16, 7)	ns, ns	1, 80, ns, 10, 15	left OFC	YBOCS, HARS, HDRS	rTMS showed a significant reduction in YBOCS scores compared to sham treatment for up to 10 weeks (*p* < 0.02), with the effect fading by 12 weeks (*p* < 0.06). Anxiety and depression symptoms also decreased, but no significant differences were found between the groups for these outcomes.
Kang et al. [61]	Randomized, single-blind, sham-controlled	20 (10, 10)	27.4 (11.6), 3:17	1, 110, 1200, 20, 10	right dlPFC+SMA	Y-BOCS, MADRS	There were no significant differences between active rTMS and sham on YBOCS (F = 0.01, *p* = 0.92) or MADRS (F = 0.39, *p* = 0.54). Time had a significant effect on both YBOCS (F = 5.48, *p* = 0.009) and MADRS (F = 6.55, *p* = 0.004), but no group-by-time interactions (YBOCS: F = 0.03, *p* = 0.94; MADRS: F = 0.09, *p* = 0.67). This suggests rTMS had no therapeutic effect on OCD symptoms.
Sachdev et al. [62]	Randomized, double-blind, sham-controlled	18 (10, 8)	32.3 (9.2), 8:10	10, 110, 1500, ns, 10	left dlPFC	Y-BOCS, the Maudsley Obsessive-Compulsive Inventory, MADRS, BDI, STAI-I	rTMS did not significantly reduce YBOCS or Maudsley Obsessive-Compulsive Inventory scores in treatment-resistant OCD patients compared to sham treatment. While a reduction in YBOCS was observed after 20 sessions, it was not significant when controlling for depression, suggesting that rTMS over the left DLPFC is ineffective for treatment-resistant OCD.
Prasko et al. [63]	Randomized, double-blind, sham-controlled	30 (18, 12)	28.9 (7.7), 18:12	1, 110, 1800, 30, 10	left dlPFC	Y-BOCS, CGI, HAM-A, BAI	rTMS did not show a significant advantage over sham treatment in enhancing the effects of serotonin reuptake inhibitors in OCD patients. Both groups improved during the study, but there was no difference between them.
Alonso et al. [64]	Randomized, double-blind, sham-controlled	18 (10,8)	32.5 (12.9), 6:12	1, 110, ns, 20, 18	right dlPFC	Y-BOCS, HAM-D	no significant improvement in OCD symptoms with 18 sessions of low-frequency rTMS over the right prefrontal cortex compared to sham treatment. While a few patients showed improvement, overall, rTMS was ineffective for OCD with the given parameters.

Abbreviation: N: Number, (I, C): (Intervention, Control), M: Male, F:Female, D: Duration, m: minutes, F: Frequency, ns: not specified, The orbitofrontal cortex (OFC), the Yale-Brown Obsessive-Compulsive Scale (Y-BOCS), the Hamilton Anxiety Rating Scale (HAM-A), the Hamilton Depression Rating Scale (HAM-D), the Sheehan Disability Scale (SDS), and the Clinical Global Impression Scale (CGI-S), Beck Depression Inventory (BDI), Beck Anxiety Inventory (BAI), the Global Assessment Function (GAF), dorsolateral prefrontal cortex (dlPFC), dorsal medial prefrontal cortex (dmPFC), Dorsal anterior cingulate cortex (dACC), Symptom Checklist 90 (SCL-90), Acceptance and Action Questionnaire (AAQ-II), and Cognitive Fusion Questionnaire (CFQ), Acceptance and Commitment Therapy (ACT), Minimental state Examination (MMSE), The Montreal Cognitive Assessment (MoCA), Brown Assessment of Beliefs Scale (BABS), 24-item Hamilton Depression Scale (HAMD-24), Inventory of Depressive Symptomatology (IDS), Hospital Anxiety Scale (HADS), Hospital Depression Scale (HRDS), Mini-Mental State Examination (MMSE), the Montgomery–Asberg Depression Rating Scale (MADRS), the Spielberger State Trait Anxiety Inventory-I (STAI-I),, Zung Self-Administered Scale (Zung-SAS), Hamilton Depression Rating Scale (HDRS), YBOCS – Self-rating (YBOCS-SR), Patient Global Impression (PGI), Dimensional Obsessive-Compulsive Scale (DOCS), Obsessive–Compulsive Inventory–Revised (OCI-R), Trail Making Test (TMT), Stroop Color and Word Test (SCWT), Digit Span Test (DS), accelerated high-dose theta burst stimulation (ahTBS), Obsessive Belief Questionnaire (OBQ44), Behavioral Avoidance Scale (BAS)

## Target regions

### Supplementary motor area (SMA)

Seventeen studies targeted the SMA or pre-SMA in OCD, most applying low-frequency 1 Hz rTMS over multiple sessions. In the bilateral SMA subgroup, several trials reported meaningful symptom reductions compared with sham, with benefits in some cases sustained for weeks or months [37, 48, 54, 65]. These improvements often extended to comorbid anxiety and depression and, in some cases, were associated with changes in brain connectivity or metabolic activity [25, 37]. However, not all results were significant and promising; for example, Guo et al. [35], found no significant improvement in OCD symptoms with continuous theta-burst stimulation, and Zou et al. [31], observed no overall advantage over psychological therapy.

For the pre-SMA, several studies also demonstrated significant benefits, including higher responder rates and sustained improvements [41, 55, 57], with genetic analyses suggesting that treatment response may depend on specific polymorphisms [41]. Other trials, however, found no advantage over sham in medication-resistant patients [47] or with theta-burst stimulation [42]. More recent work has moved toward individualized protocols: Chu et al. [20], identified genetic markers predicting responsiveness, Ji et al. [38], showed personalized targeting improved both symptoms and connectivity patterns, and Jiang et al. [28], reported that accelerated high-dose TBS yielded short-term effects similar to 1 Hz stimulation.

### Dorsolateral prefrontal cortex (dlPFC)

Fourteen studies targeted the dlPFC. It is crucial to note that studies explored different protocols, specifically examining laterality, as the right and left dlPFC are thought to exert distinct modulatory effects on the CSTC circuit. Four studies applied bilateral dlPFC stimulation, mostly using high-frequency rTMS. Several reported significant reductions in OCD severity, with some also noting improvements in cognitive performance [45, 50, 51, 53]. Benefits were observed particularly in treatment-resistant patients, and one study showed sustained anxiety and OCD improvements at follow-up Ma et al. [53]. Five trials targeted the left dlPFC, with mixed results. Some found significant benefits, particularly when rTMS was used as an adjunct to pharmacotherapy [26, 58], while others reported no significant differences from sham [33, 62, 63]. Variability in outcomes may relate to differences in stimulation frequency, intensity, and patient characteristics. Right dlPFC stimulation was also examined in five studies. While some reported improvements in OCD symptoms and mood [46, 49], others found no significant differences from sham [56, 59, 64]. Interestingly, Elbeh et al. [49], observed better clinical outcomes with low-frequency (1 Hz) compared to high-frequency stimulation.

### Combined dlPfC and Sma/pre-SMA stimulation

Five studies investigated protocols targeting both the dlPFC and SMA or pre-SMA, often including additional sites such as the vertex. Most applied high-frequency rTMS (10 Hz) with 16 sessions. Several reported meaningful symptom reductions, particularly when combined with exposure and response prevention (ERP) therapy [27] or when treatment response was linked to specific activation patterns in planning, inhibition, and emotional processing networks [21, 22]. Neuroimaging findings indicated that greater pre-treatment activation in regions such as the right amygdala and dorsomedial prefrontal areas predicted better outcomes, while reduced activation in error-processing networks was associated with symptom improvement [21, 22]. One trial combining rTMS and ERP found decreased activation in planning and error-processing regions but no significant differences in symptom reduction between treatment protocols [24]. In contrast, a low-frequency (1 Hz) right dlPFC + SMA protocol showed no therapeutic effect on OCD symptoms compared with sham [61].

### Orbitofrontal cortex (OFC)

Five studies investigated rTMS targeting the OFC, either unilaterally or bilaterally. Left OFC stimulation showed mixed results: one trial using intermittent theta-burst stimulation reported a significant group × time interaction for OCD symptoms, but the effect was only maintained for certain secondary measures when controlling for other variables [39], while another found significant and lasting reductions in OCD severity along with improvements in anxiety and depression [60]. Right OFC stimulation produced less consistent effects. Zıblak et al. [40], reported no significant differences from sham, whereas Nauczyciel et al. [52], observed symptom reductions and PET evidence of decreased OFC metabolism after low-frequency rTMS, although clinical improvement did not reach statistical significance. Also, one study stimulating both the right dlPFC and right OFC and found no significant differences between active rTMS and sham on YBOCS [61].

### Anterior cingulate cortex (ACC)

Four studies targeted the ACC, either directly or in combination with the dorsomedial prefrontal cortex (dmPFC). A single trial of dACC stimulation reported significant and sustained OCD symptom improvement, with added gains in cognitive performance such as response monitoring and error correction [36]. Three other studies applied high-frequency rTMS to the dmPFC combined with ACC stimulation. One trial found a significant reduction in Y-BOCS and HAM-A scores with double-cone coil stimulation, although symmetry-related symptoms did not improve significantly [23]. Two large, pivotal trials by (Carmi et al. [16, 43], which utilized the H7 Deep TMS (dTMS) coil targeting the mPFC + ACC, demonstrated robust clinical benefits. In the 2019 study, response rates (30% Y-BOCS reduction) were 38.1% in the active group versus 11.1% in the sham group, along with significant CGI-I and CGI-S improvements. These findings provided the core evidence supporting the FDAs clearance of this specific dTMS protocol for OCD. Symptom provocation before each session and changes in error-related negativity theta activity were associated with clinical gains [43].

## Stimulation frequency

### 1 Hz stimulation

Low-frequency (1 Hz) rTMS was the most frequently investigated protocol, examined across multiple cortical targets including the SMA, pre-SMA, dlPFC, OFC, and ACC. Several studies targeting the bilateral SMA or pre-SMA reported significant and lasting reductions in OCD symptoms [25, 34, 36–38, 41, 48, 54, 55, 57], with some also showing improvements in anxiety, depression, and cognitive performance. Neurophysiological changes, such as modulation of motor cortex excitability [54], normalization of hemispheric asymmetry [57], and altered functional connectivity [37, 38], were linked to clinical gains. However, several trials reported no advantage over sham, particularly in medication-resistant populations [33, 40, 44, 47, 61, 63, 64], or when paired with active psychotherapies where both groups improved [31]. Mixed results were also seen in OFC stimulation: while left OFC stimulation reduced symptoms for up to 10 weeks [60], right OFC studies often failed to show significant effects [40, 52].

Positive findings were also observed for left dlPFC stimulation in treatment-refractory OCD when used adjunctively with medication [26] and for right dlPFC in reducing OCD and depression scores [46]. In contrast, other dlPFC studies showed no significant differences from sham [33, 63, 64].

### 10 Hz stimulation

High-frequency 10 Hz rTMS has been explored across several cortical targets, most commonly in combined dlPFC and pre-SMA protocols. Four studies applied 10 Hz stimulation to these sites, often alongside exposure and response prevention. Significant OCD symptom reductions were reported in multiple trials [24, 27], with response rates exceeding 50% in some cases. Neuroimaging findings indicated that pretreatment activation and connectivity patterns such as lower precuneus activation in dlPFC stimulation and lower insular-amygdalar connectivity in pre-SMA stimulation were associated with better outcomes [21, 22]. These studies also linked symptom improvement to decreased activation in planning- and error-processing networks [24, 27]. However, results were not uniformly positive. Trials targeting the left or right dlPFC alone reported no significant OCD improvements compared with sham [56, 59, 62]. While some of these studies observed modest gains in mood measures such as depression or anxiety, these effects did not extend to core OCD symptoms.

### 20 Hz stimulation

High-frequency 20 Hz rTMS was examined in both dlPFC- and mPFC/ACC-targeted protocols. Three studies targeting the bilateral dlPFC reported significant OCD symptom reductions compared with sham [45, 50, 51]. Improvements in cognitive performance such as verbal processing speed, flexibility, and executive functioning were noted in some trials [50], while others found no cognitive effects despite symptom relief [45]. One trial found that full and partial clinical responses occurred only in the active rTMS group [51].

Left dlPFC stimulation alone did not yield significant effects as monotherapy but showed clinical benefit when used adjunctively with SSRIs in patients with poor medication response [58]. Deep TMS (dTMS) studies targeting the mPFC and ACC using 20 Hz also showed consistent symptom reductions. Ozer et al. [23], reported significant decreases in Y-BOCS and HAM-A scores, with a 35% response rate versus 6.7% in sham. Two trials by Carmi et al. [16, 43], demonstrated response rates of 38–45% in active groups compared with 7–11% in sham, along with significant CGI-I and CGI-S improvements. Symptom provocation before each session and changes in error-related negativity theta activity were associated with clinical gains [43].

### Other frequencies and theta-burst stimulation (TBS)

A small number of studies examined alternative stimulation frequencies and patterned protocols. Elbeh et al. [49], directly compared 1 Hz and 10 Hz rTMS over the right dlPFC, finding that 1 Hz produced significantly greater improvements in OCD and anxiety symptoms than both sham and 10 Hz. Ma et al. [53], used an individualized alpha EEG-guided rTMS protocol (8 × 12 Hz) over the bilateral dlPFC, reporting significant and sustained reductions in OCD and anxiety, with depression improvements emerging later, likely as a secondary effect of core symptom relief.

Several clinical trials investigated continuous theta-burst stimulation (cTBS; 50 Hz bursts at 5 Hz). Dutta et al. [39], applied cTBS to the left OFC, finding a significant group × time interaction for OCD, anxiety, and depression scores, but when adjusting for anxiety and depression changes, only HAM-A and CGI-S remained significant. Guo et al. [35], applied cTBS to the bilateral SMA and found no OCD symptom benefit, though short-term mood improvements were observed. Harika-Germaneau et al. [42], delivered cTBS to the pre-SMA and reported no significant differences from sham for OCD or secondary outcomes. Accelerated high-dose TBS (ahTBS) to the right pre-SMA was tested by Jiang et al. [28], producing significant OCD symptom reductions over five days, comparable to 1 Hz rTMS, with a non-significant trend toward higher responder rates in the ahTBS group. Overall, both the targeted brain region and stimulation frequency are critical determinants of rTMS efficacy in OCD. SMA and pre-SMA respond best to low-frequency 1 Hz, while dlPFC outcomes improve with multi-target or combined behavioral approaches. mPFC/ACC deep TMS at 20 Hz produces some of the strongest and most consistent effects. Personalized targeting, biomarker-informed protocols, and integration with psychotherapy may further enhance treatment outcomes.

## Discussion

In this systematic review, we critically evaluated the current clinical evidence on the efficacy of rTMS as a therapeutic option for OCD. Overall, findings from the 47 included studies indicated a heterogeneous clinical response, highlighting the complexity and variable efficacy of rTMS in treating OCD. The majority of included studies targeted the dlPFC and the SMA, consistent with previous findings implicating dysfunction in these cortical areas within OCD pathophysiology [7, 8]. Particularly, rTMS targeting the SMA frequently demonstrated favorable clinical outcomes, which aligns with the neurobiological understanding of SMAs role in inhibitory control and motor-related compulsive behaviors in OCD [25, 37, 38]. Furthermore, consistent improvements in OCD symptomatology following SMA stimulation across several RCTs (e.g. [34, 55]) indicated its relevance as a critical neural target for therapeutic intervention. The demonstration of clinical efficacy, particularly by Deep TMS targeting the mPFC/ACC [16], which led to FDA clearance in 2018, has fundamentally shifted the primary research question from ‘Does rTMS work?’ to ‘Which rTMS protocol, targeting which circuit, is most effective for a given patient?’ Our expanded analysis addresses this key post-clearance research need by examining and comparing the efficacy of the FDA-cleared dTMS target alongside traditional targets like the dlPFC and SMA, and incorporating new predictive biomarkers.

Notably, low-frequency (1 Hz) rTMS over both SMA and dlPFC demonstrated significant clinical improvements, including reductions in OCD severity scores, anxiety, and depressive symptoms [21, 22, 24]. These beneficial outcomes are likely related to the modulation of cortical excitability within dysfunctional cortico-striato-thalamo-cortical (CSTC) networks, known to underlie OCD symptoms [66]. Conversely, other studies failed to report significant therapeutic benefits of rTMS when targeting these areas [44, 47], underscoring the existence of variability and complexity in treatment response.

Several studies explored innovative methodological integrations, combining rTMS with pharmacological and psychotherapeutic interventions [14, 67, 68]. For instance, the combined modality of rTMS, exposure and response prevention, and biofeedback yielded stable and pronounced reductions in OCD symptoms and anxiety, suggesting potential synergistic therapeutic effects when combining neurostimulation with established therapeutic methods [24, 68]. However, definitive conclusions on superiority remain premature due to the paucity and heterogeneity of such combinatorial studies.

## Comorbidity, pathophysiological overlap, and clinical correlates

A critical clinical consideration is the high rate of comorbidity, particularly anxiety and depression, which frequently co-occur with OCD. Our findings indicate that rTMS protocols showing efficacy in OCD (e.g., 1 Hz over SMA, 20 Hz dTMS over mPFC/ACC) often lead to concurrent improvements in these accompanying anxiety and depressive symptoms [23, 24, 46]. This observed co-amelioration raises the possibility of a pathophysiological overlap; rTMS might be modulating common fronto-limbic circuits (such as the CSTC circuits and the medial prefrontal cortex) that are implicated in the regulation of all three conditions. For instance, the dlPFC is a well-established target for rTMS in major depressive disorder, and its stimulation may reduce OCD severity partly by alleviating comorbid depression. Discussing this possible dual benefit is clinically vital, but it also necessitates caution, as some studies found OCD improvement independent of mood changes. Future research should use statistical models to disentangle the direct effect of rTMS on core OCD symptoms from its secondary effects mediated by the amelioration of comorbid affective symptoms.

## Regulatory context: benchmarking against depression

The success of rTMS in OCD should be viewed in the context of its established role in treating major depressive disorder. rTMS is a globally approved therapy for treatment-resistant depression, with regulatory clearance and public health reimbursement established in key jurisdictions, including the USA (FDA-cleared), Germany, Australia, Canada, and France. This widespread acceptance of rTMS for MDD is significant for OCD, as it establishes a robust infrastructure (e.g., equipment, trained personnel, and reimbursement pathways) that can facilitate the clinical uptake of the recently FDA-cleared dTMS protocol for OCD. Moreover, the long-standing use of rTMS over the DLPFC for depression lends weight to the theoretical and clinical plausibility of targeting similar frontal networks to manage comorbid OCD and anxiety.

In the reviewed studies, significant predictors of treatment response to rTMS were also identified, highlighting the potential role of personalized medicine approaches. Neuroimaging findings indicated that pretreatment neural activation patterns, particularly amygdala and precuneus activity, could predict therapeutic outcomes, suggesting these neural biomarkers could be utilized to optimize patient selection and enhance clinical outcomes [21, 22]. Additionally, genetic polymorphisms (e.g., RS4680, RS16965628, RS1019385) were identified as potential biological predictors of response to rTMS and selective serotonin reuptake inhibitors (SSRIs) [20], further supporting the importance of precision medicine approaches in OCD treatment. Despite these encouraging findings, substantial methodological limitations were noted across included studies. A considerable number of studies demonstrated unclear or high risk of bias, particularly regarding randomization procedures, allocation concealment, and blinding, potentially influencing outcomes and reducing reliability [24, 32, 37]. Additionally, the variations in rTMS parameters, such as frequency, intensity, number of sessions, and targeted brain regions, significantly limit the comparability across studies and hinder definitive conclusions regarding optimal stimulation protocols.

## Limitation and suggestion

This review has several limitations inherent to systematic reviews, including the exclusion of non-English publications and grey literature, which may have introduced publication bias and led to the omission of relevant evidence. Despite comprehensive database searches, unpublished data or ongoing trials may further inform the efficacy of rTMS in OCD. Additionally, variability in reporting clinically relevant variables across studies constrained deeper analyses. While stimulation parameters such as frequency, intensity, pulses, and session duration were systematically extracted, other important factors including precise stimulation site localization, duration of illness, medication history (e.g., antidepressant use), and degree of treatment resistance were often missing. Variability in treatment resistance, in particular, may have influenced responsiveness and contributed to outcome heterogeneity.

Several methodological challenges were also evident. A substantial proportion of trials demonstrated unclear or high risk of bias in randomization and blinding (e.g., 36% for random sequence generation [32, 45]), compounded by the inherent difficulty of maintaining participant blinding in non-invasive stimulation studies. Considerable heterogeneity in stimulation protocols including frequency (1 Hz to 20 Hz and TBS), number of sessions (5–30), and targeted regions (SMA, dlPFC, OFC, ACC) further limited comparability and hindered the development of standardized therapeutic guidelines. Moreover, the absence of consistent long-term follow-up across studies restricted conclusions regarding durability of treatment effects [28, 31]. While some SMA-targeted trials reported sustained benefits lasting several months [37, 65], the lack of standardized maintenance protocols prevents firm conclusions. The varied use of rTMS as monotherapy versus an adjunctive intervention further complicates interpretation of its absolute efficacy [26, 47].

To overcome these limitations, future research should focus on rigorously designed, multi-center RCTs with standardized stimulation parameters, robust blinding, and comprehensive reporting of clinical characteristics, including illness duration, treatment history, and resistance profiles. Incorporating long-term follow-up is essential to assess durability and determine the role of maintenance protocols. Additionally, combined approaches integrating rTMS with pharmacological or psychotherapeutic treatments, particularly ERP, may enhance efficacy and broaden clinical applicability. Biomarker-informed studies emphasizing individualized targeting are warranted to identify predictors of treatment response and refine patient selection. The strongest evidence currently supports 1 Hz stimulation over the SMA and 20 Hz dTMS targeting the mPFC/ACC (the only FDA-cleared rTMS protocol for OCD), with frequent concurrent improvements in comorbid anxiety and depression [24], underscoring the potential of rTMS as both an adjunctive and alternative treatment for OCD. Future research should prioritize comparative effectiveness trials directly contrasting the FDA-cleared dTMS protocol against high-efficacy conventional rTMS protocols (e.g., SMA targeting) to establish a clear optimal clinical pathway. A key contribution of our work is the detailed assessment of methodological heterogeneity inherent in the rTMS literature. Our systematic analysis of 47 RCTs allowed for highly granular observations, such as the mixed efficacy of 1 Hz stimulation across different target regions (SMA vs. dlPFC) and the identification of studies failing to report crucial parameters like treatment resistance profiles. Although most prior meta-analyses noted heterogeneity, our expanded dataset allows for a more definitive identification of the most promising protocols (e.g., 1 Hz over SMA; 20 Hz dTMS over mPFC/ACC) and confirms that methodological gaps in blinding and parameter reporting remain the most critical barriers to standardizing rTMS treatment.

Moreover, the absence of consistent long-term follow-up across studies restricted conclusions regarding the durability and sustainability of treatment effects [28, 31]. While the effectiveness of rTMS in the acute phase is becoming clearer, the sustainability of response over several months is the key determinant of its clinical utility and necessity for maintenance treatment. While some SMA-targeted trials reported sustained benefits lasting several months [37, 65], the lack of standardized maintenance protocols prevents firm conclusions on long-term benefit. Future high-quality RCTs must incorporate consistent follow-up assessments (e.g., 3, 6, and 12-months post-treatment) to allow for a robust meta-analytic synthesis of the long-term efficacy and to determine the necessity and characteristics of maintenance rTMS protocols.

Variability in treatment resistance, in particular, may have influenced responsiveness and contributed to outcome heterogeneity. Furthermore, the varied use of rTMS as monotherapy versus an adjunctive intervention significantly complicates interpretation of its absolute efficacy. Our included trials demonstrated that rTMS was employed in three primary ways: **1)** Monotherapy: rTMS administered to drug-naïve patients or those after medication washout (e.g. [34]); **2)** Adjunctive to pharmacotherapy: rTMS added to patients already on stable doses of SSRIs or antipsychotics (e.g. [26, 58]); and **3)** Adjunctive to psychotherapy: rTMS combined with exposure and response prevention (ERP) (e.g. [24]). The efficacy of rTMS often appears highest when used as an augmentation strategy, suggesting a synergistic effect with existing treatments. Future research and reporting must clearly delineate the use case (monotherapy vs. adjunctive) to establish its precise role in the treatment cascade.

## Conclusion

This systematic review synthesizes results of prior research together with the existing clinical evidence regarding the therapeutic efficacy of rTMS for OCD. Although the results across studies are heterogeneous, rTMS demonstrates potential as an effective adjunctive treatment, particularly when targeting regions such as the SMA and dlPFC. Evidence also supports its consideration as an alternative monotherapy in select populations, such as drug-naïve patients. Significant variability in outcomes underscores the necessity of standardizing stimulation protocols and enhancing methodological rigor in future trials. Integrative therapeutic strategies combining rTMS with pharmacological and psychotherapeutic interventions may further enhance treatment efficacy. Additionally, leveraging neuroimaging and genetic biomarkers to personalize rTMS treatment holds promise for improving therapeutic outcomes. Overall, while promising, definitive conclusions regarding the optimal use of rTMS in clinical practice await further high-quality research.

## Data Availability

All data generated or analyzed during this study are included in this published article.
